# Development and application of a triplex ddPCR method for simultaneous detection of MLSB resistance genes in feces and environmental samples

**DOI:** 10.3389/fmicb.2026.1887402

**Published:** 2026-08-05

**Authors:** Yumei Guo, Xirong Yin, Hong Zhang, Xinyan Dong, Huifang Tian, Ke Wang

**Affiliations:** 1 Shijiazhuang Center for Disease Control and Prevention, Shijiazhuang, China; 2 School of Public Health, Hebei Medical University, Shijiazhuang, China; 3 Hebei Key Laboratory of Intractable Pathogens, Shijiazhuang, China; 4 Shijiazhuang Technology Innovation Center for Chemical Poison Detection and Risk Early Warning, Shijiazhuang, China

**Keywords:** antibiotic resistance genes, *erm* genes, MLS_B_ resistance genes, triple detection, triplex droplet digital PCR

## Abstract

Macrolide-lincosamide-streptogramin B (MLS_B_) resistance has emerged as a critical public health challenge, significantly reducing therapeutic efficacy and exacerbating healthcare burdens. Therefore, the establishment of advanced molecular diagnostic techniques for the identification of MLS_B_ resistance genes is critically important. Such techniques not only offer precise guidance for clinical therapy, but also provide a robust foundation for the development of effective epidemiological surveillance strategies. In this study, a novel droplet digital PCR (ddPCR) method was developed that enables the simultaneous detection and absolute quantification of three MLS_B_ resistance genes (*ermB*, *ermF*, and *ermG*). Through rigorous design and validation of primers and probes, combined with systematic optimization of ddPCR parameters, including annealing temperature, concentration of primer/probe sets and extension time, the optimized method was able to detect single-copy *erm* genes in samples. Additionally, the developed triple ddPCR method demonstrated excellent sensitivity, with limits of detection (LOD) ranging from 3.37 to 8.31 copies per reaction, and good repeatability (coefficient of variation < 20%), adequately meeting the requirements for accurate quantification of *erm* genes. Furthermore, this new method was applied to a total of 137 samples, including 18 human fecal samples, 40 animal-derived food samples, 20 sewage samples, 35 surface water samples and 24 farm-derived samples. Among them, 97.08% detected positive for *ermB*, 91.97% for *ermF*, and 88.32% for *ermG,* with *erm* gene concentrations ranging from undetectable to 4.79 × 10^9^ copies/g. In summary, this novel ddPCR method enables efficient detection and quantification of MLS_B_ resistance genes, providing valuable support for antimicrobial resistance surveillance and evidence-based infection control measures.

## Introduction

Macrolides, lincosamides, and streptogramin B share overlapping mechanisms of action, and are therefore collectively classified as MLS_B_ antibiotics. These agents are widely used in clinical practice to treat infections caused by Gram-positive bacteria and anaerobic bacterial (Marosevic et al., 2014). However, pathogens carrying MLS_B_ resistance genes may become cross-resistant to all three classes of antimicrobials, rendering therapeutic regimens ineffective. Quezada-Aguiluz et al. (2022) found that 97% of methicillin-resistant *Staphylococcus aureus* (MRSA) isolates from Chilean hospitals exhibited MLS_B_ resistance.

Traditional methods for detecting MLS_B_ resistance primarily rely on bacterial culture and antimicrobial susceptibility testing (AST). Although these approaches remain valuable in clinical diagnostics, they are inherently limited by their time-consuming nature (typically requiring 2–3 days), low sensitivity, and inability to detect non-culturable or slow-growing microorganisms (Barbanti et al., 2016). Moreover, such phenotypic assays fail to distinguish between intrinsic and acquired resistance mechanisms, thereby limiting the understanding of the molecular basis of resistance development and transmission. With advances in molecular biology techniques, genotypic detection of ARGs (Antibiotics resistance gene) has emerged as a more efficient and informative alternative. Polymerase chain reaction (PCR)-based assays, including conventional PCR, real-time quantitative PCR (qPCR), and next-generation sequencing, have been widely applied to identify and quantify ARGs in complex microbial communities. However, these methods often suffer from limitations such as high background noise, poor reproducibility, and reliance on standard curves for quantification, which can compromise the accuracy and precision of gene copy number estimation - especially for low-abundance targets (Duan et al., 2025).

Droplet digital PCR (ddPCR), a third-generation PCR technology, offers a promising solution to these challenges. Unlike qPCR, ddPCR partitions the sample into tens of thousands of nanoliter-scale droplets, each serving as an independent amplification reaction (Fortunato et al., 2018). Through end-point PCR and Poisson distribution-based statistical analysis, ddPCR enables absolute quantification without the need for standard curves, achieving a detection limit as low as several copies per microliter. This high sensitivity, combined with improved precision and reproducibility, makes ddPCR particularly suitable for applications requiring accurate quantification of low-abundance genetic targets, such as rare mutations, viral load measurements, and ARG surveillance. Multiplex ddPCR further enhances its utility by enabling simultaneous detection of multiple target sequences in a single reaction. Various strategies have been developed to achieve multiplexing, including probe fluorescence intensity differentiation and dual-labeled fluorescent probes. These approaches allow researchers to investigate complex microbial profiles and co-occurrence patterns of multiple ARGs, offering insights into resistance gene dynamics and potential horizontal gene transfer events (Huang et al., 2024).

Molecular methods are increasingly used in clinical and environmental settings, however, under the concept of “One Health,” healthy people are also potentially important sources of infection for carrying pathogens and transmitting resistance genes (Zeng et al., 2023). The human gut microbiota has become an important repository of ARGs (Sommer et al., 2010). Existing studies predominantly concentrate on antibiotic resistance profiles in clinical cohorts, with healthy populations only serving as routine controls. Notably, few studies have systematically investigated intestinal antibiotic-resistant bacteria and resistance genes across the combined cohort of pregnant women, children, and pharmaceutical workers. To fill this research gap, we enrolled these three populations to further validate the applicability of our established digital PCR assay.

Among the diverse types of MLS_B_ resistance genes, the *erm* gene family is the most extensively studied and widely distributed. Genes such as *ermA*, *ermB*, *ermC*, *ermF*, and *ermG* encode 23S rRNA methyltransferases that modify ribosomal binding sites, conferring resistant to macrolides, lincosamides, and streptogramin B antibiotics (Tan et al., 2024). *ermB* and *ermF* are frequently detected in environmental and clinical samples (Che et al., 2019; Wang Y. T. et al., 2022), whereas ermG remains rarely reported due to its narrow host range (Letchumanan et al., 2016); however, all three genes can be carried by integrative and conjugative elements (ICEs) with high dissemination potential (Che et al., 2019), warranting simultaneous quantitative detection in target environments. Despite their importance, few studies have developed highly sensitive and specific multiplex ddPCR assay for simultaneous quantification of *ermB*, *ermF*, and *ermG*. Most existing methods either focus on single-gene detection or lack sufficient validation in terms of specificity, sensitivity, and reproducibility. Therefore, there is a critical need for a robust, standardized approach to quantify these key resistance determinants across diverse sample types.

This work contributes to the growing field of molecular tools for antibiotic resistance monitoring and provides a reliable methodology for tracking the dissemination of clinically and environmentally relevant MLS_B_ resistance genes (*ermB*, *ermF*, and *ermG*). By integrating ddPCR technology with a ratio-probe strategy, and through the rational design of primers and probes along with the optimization of key PCR parameters, a novel and highly specific triple ddPCR assay was successfully established. The new method enhancing the sensitivity and efficiency of *erm* gene detection. This method detects erm resistance genotypes at an early stage, monitors treatment efficacy dynamically, and evaluates contamination from resistance genes strictly. Establishing a robust quantitative assay for *erm* genes extends the utility of ddPCR in ARG monitoring and enhances insights into their ecological prevalence, offering critical technical support for assessing the risks associated with *erm*-mediated resistance.

## Materials and methods

### Sample collection and bacterial isolation

**Figure 1:**
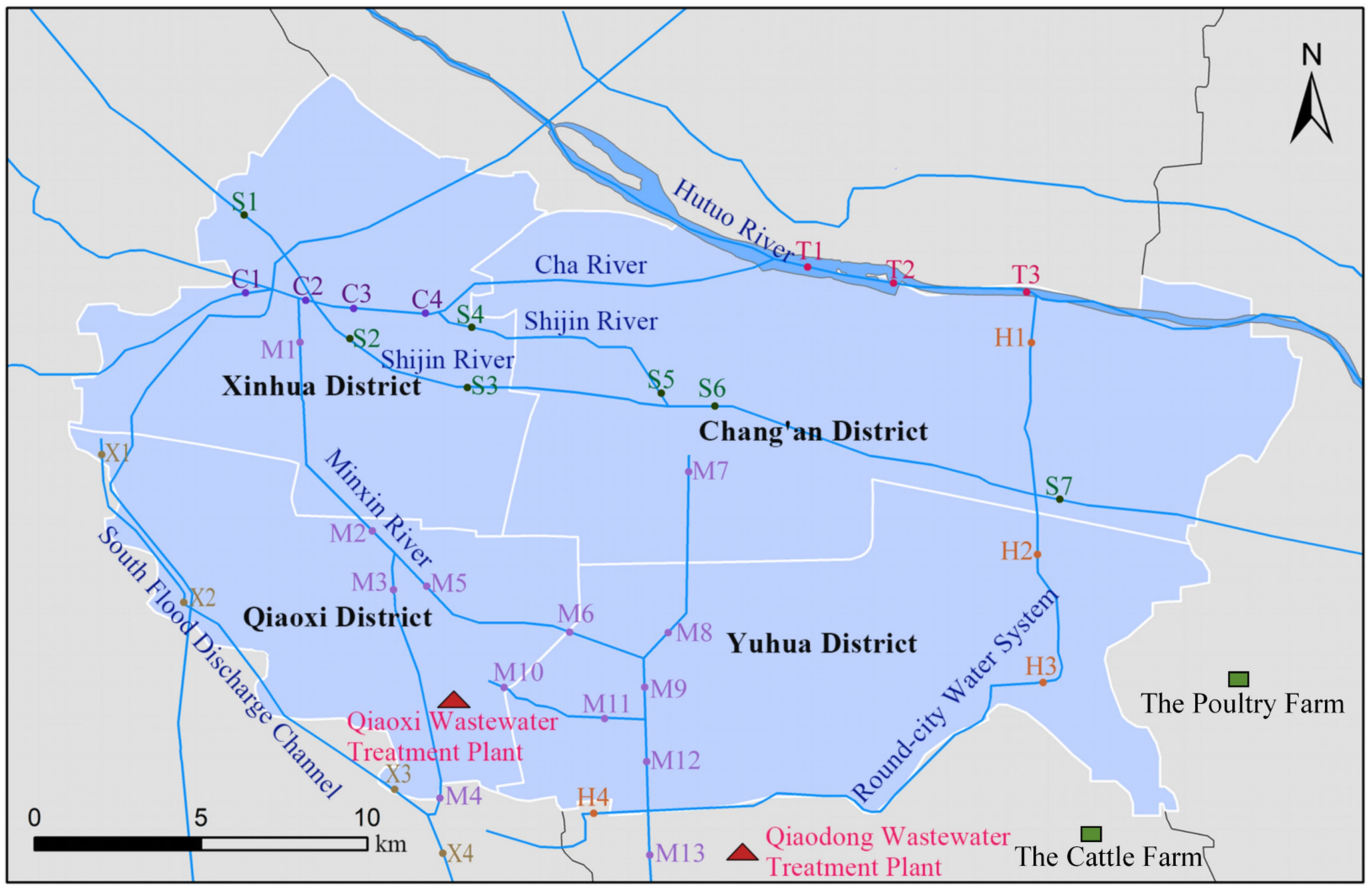
Sampling point locations in Shijiazhuang. C, Cha River; H, Round-city water system; M, Minxin River; S, Shijin River; T, Hutuo River; X, South Flood Discharge Channel.

To comprehensively validate the practical applicability of the method, 137 samples including environmental, food, and human fecal samples were collected locally from Shijiazhuang, China, for subsequent analysis. Subsequently, drug-resistant bacteria were isolated and cultured from these samples using tryptic soy agar medium supplemented with antibiotics. Antimicrobial susceptibility testing was then performed, leading to the identification of nine resistant bacterial strains. A total of 137 samples were collected from various sources. A total of 20 fecal samples from healthy humans. Forty food samples consisted of meats (fish, shrimp, pork, chicken) and internal organs (liver, heart), which were purchased from markets. Eighteen sewage samples were obtained from 2 wastewater treatment plants servicing 90% of the central urban region. Thirty-five surface water samples were collected from 6 rivers in Shijiazhuang city, and the sampling sites effectively covered urban areas with a high population density. Additionally, 24 samples were collected from a small-scale poultry farm and a cattle farm, including soil, animal feces, drinking water, feed, poultry eggs and raw milk. These samples represented diverse environmental and biological matrices associated with livestock husbandry practices. The sampling points for surface water, wastewater treatment plants and two farms are showed in Figure 1 (Yin et al., 2025). Samples used for method validation and formal experiments were both collected between May and October 2024 from the same market, wastewater treatment plant and aquatic environment. All samples were placed in disposable sterile containers and conveyed to the laboratory for pretreatment within 4 h. Samples were preserved at a temperature ranging from 4 to 8 °C. Human biological specimens utilized in this study were approved by the Ethics Committee of the Shijiazhuang Center for Disease Control and Prevention (Approval No. 2022–002).

### Preprocessing of samples and DNA extraction

The pretreatment operations for animal-derived food samples, sewage samples, and surface water samples were as follows: Under aseptic conditions, accurately weighed 25 g of food samples and added them to a container containing 225 mL of physiological saline. Then homogenized the mixture thoroughly using a homogenizer (AngniInstrument, China). A total of 10 mL of the homogenate was transferred to a centrifuge tube and centrifuged at 8,000 r/min for 10 min. After centrifugation, carefully discarded the supernatant. A total of 200 μL of normal saline was added to the centrifuge tube. Subsequently, pipetted up and downed to resuspend the precipitate, which will be used as the sample for DNA extraction. Untreated sewage exhibits colloidal properties with high particulate loads, for which 0.45 μm membrane filtration is recommended (Lu et al., 2015), whereas 0.22 μm membranes were used for surface water samples to effectively enrich microorganisms (Lu et al., 2019). After that, the used filters were stored at −80 °C until DNA extraction was performed.

Total DNA of sewage and surface water samples was extracted by the DNeasy PowerWater Kit (Qiagen, Germany). Power soil DNA Kit (Qiagen, Germany) was employed for the DNA extraction of animal-derived soil samples. Total DNA were extracted from all pre-treated food samples, as well as milk and egg samples collected from farms, using the Tianlong Bacterial DNA Extraction Kit (Tianlong, China). PowerFecal Pro DNA Kit (Qiagen, Germany) was employed for the DNA extraction of fecal samples from farms and human (Faria et al., 2023; Künz et al., 2025), with approximately 200 mg of each sample used. A unified lysis protocol was applied to all samples following preprocessing. Eventually, the concentration and quality of DNA were determined via a NanoDrop 2000 spectrophotometer (Thermo Scientific, United States). All DNA samples were stored at −20 °C until further use.

### Design and validation of primers and probes

Specific primers and probes for *ermB*, *ermF,* and *ermG* gene were designed using Oligo7.6 software (Molecular Biology Insights Inc., USA) to ensure optimal amplification efficiency and specificity. Nucleotide BLAST was employed to preliminarily assess the specificity of amplicons. In addition, the primer and probe sequences along with relevant informations for triple ddPCR are listed in Table 1, and all primer and probe sets used in this experiment were synthesized by Takara Biotech (Beijing, China).

**Table 1:** Primer and probe sequences for ddPR PCR of the *erm* genes.

Genes	NCBI number	Primers/probes	Sequence (5′–3′)	Product size (bp)	Reference
*ermB*	AF242872.1; 2,132–2,878	*ermB-*F	ACGGCCAACACAATAGGT	83 bp	This study
		*ermB-*R	GGATTCTACAAGCGTACCTTGGA		
		*ermB-*P	FAM-CACTAGGGTTGCTCTTGCACACTCAAGTC-BHQ1		
*ermF*	M17124.1; 1,182–1982	*ermF-*F	GAATTTTGCAGTTCCGAA	80 bp	This study
		*ermF-*R	GGATTTTGAAAATATCGGAAG		
		*ermF-*P	HEX-ATGCCATAAGGAATATTTGACACC-BHQ1		
*ermG*	L42817.1; 202–936	*ermG-*F	ACATCTTTGAAATAGGTGC	86 bp	This study
		*ermG-*R	ATCAATTTCTATCGCCGTA		
		*ermG-*P	HEX-AAGGTCATTTTACTGCTGAATTGG-BHQ1		
			FAM-AAGGTCATTTTACTGCTGAATTGG-BHQ1		

F, forward primer; R, reverse primer; P, probe; FAM, carboxyfluorescein; HEX, hexachlorofluorescein; BHQ1: black hole quencher 1.

To further validate the specificity of method, several strains were isolated from pig liver, chicken heart, fish viscera and sewage samples. Their resistance phenotypes were determined through antibiotic sensitivity testing. Subsequently, the bacterial nucleic acids of strains were extracted using the boiling method. Finally, the *erm* genes of 20 strains were detected simultaneously by PCR, qPCR and ddPCR (PCR and qPCR details are provided in Supplementary Tables 1,2).

### Procedure of the triple ddPCR method

The triplex ddPCR reaction mixture had a total volume of 20 μL and consisted of the following components: 10 μL of 2 × ddPCR Supermix for Probes (Bio-Rad, United States), 0.6 μL each of forward and reverse primers for all three target genes (final primer concentration: 20 μM), 0.4 μL of *ermB* probe (FAM-labeled, 20 μM), 0.4 μL of *ermF* probe (HEX-labeled, 20 μM), and 0.3 μL of dual-labeled *ermG* probes (one FAM-labeled and one HEX-labeled, both at 20 μM). In addition, 2 μL of DNA template and 3 μL of nuclease-free water were added to complete the reaction mix. The prepared 20 μL reaction mixture was thoroughly vortexed and transferred to the middle row (sample wells) of a DG8 cartridge (Bio-Rad, United States). Then, 70 μL of droplet generation oil was added to the oil wells (first row) of the same cartridge. The cartridge was then loaded into a QX200™ droplet generator (Bio-Rad, United States) to partition the reaction mixture into approximately 20,000 uniform water-in-oil droplets, with a final droplet volume of around 40 μL. The generated droplets were then carefully transferred to a 96-well PCR plate (Bio-Rad, United States), which was sealed with heat-sealing aluminum foil using a PX1™ PCR plate sealer (Bio-Rad, United States). The sealed plate was subsequently placed into a C1000™ Touch Thermal Cycler (Bio-Rad, United States) for amplification under the following thermal cycling conditions: initial at 95 °C for 10 min; 40 cycles of 94 °C for 30 s (denaturation) and at 52 °C for 60 s (annealing/extension); final enzyme deactivation at 98 °C for 10 min. The ramp rate was set to 1.5 °C/s for all steps. After amplification, the PCR plate was incubated at 4 °C for at least 30 min to stabilize the droplets. Finally, the plate was loaded into the QX200™ droplet reader (Bio-Rad, United States) for signal acquisition. Each droplet was individually analyzed for fluorescence intensity using two detection channels (FAM and HEX). Data analysis was conducted using QuantaSoft™ Analysis Pro software (version 1.0.596, Bio-Rad, United States).

### Limits of detection, dynamic range, and repeatability

The reference sequences of e*rmB*, *ermF,* and *ermG* were separately inserted into four pMD19-T plasmids without other exogenous genes. All plasmids were designed and synthesized by Takara Biotech (Beijing, China). The concentrations of the four linearized plasmid solutions used in this study were 5 × 10^8^ copies/μL. To prepare a mixed plasmid solution with a concentration of 5 × 10^7^ copies/uL, 10 μL of each of the 4 plasmid solutions was mixed with 60 μL of nuclease-free water. This solution was then serially diluted 10-fold, yielding concentrations ranging from 10^0^ to 10^7^ copies/μL. Subsequently, the gradient diluted plasmid solution was detected by triple ddPCR (3 repetitive experiments with 3 technical replicates each, *n* = 9), the average copy number, standard deviation, and coefficient of variation were calculated. To determine the reliable dynamic quantification range of triple ddPCR, quantitative curves of *erm* genes were constructed, log_10_(theoretical concentration of the standards) as the x-axis and log_10_(copies/reaction by ddPCR) as the y-axis, the linear fitting coefficient (R^2^) was calculated by Origin 2024 (OriginLab, United States). Referring to the guidelines of the Clinical and Laboratory Standards Institute (CLSI), the LOD and 95% confidence interval (95% CI) of the ddPCR were estimated by the Probit approach, and the analysis was executed by SPSS 21.0.

### Estimation of the concentration of *erm* genes in diverse samples

It must be pointed out that the concentration data obtained from ddPCR merely stand for the concentration of *erm* genes in 2 μL of the template DNA. Therefore, the following formula was developed to calculate the actual concentration.


$$C=N_{\text{mean}}\times \frac{V_{1}}{V_{2}\times B\times d}\times M$$

C represents the copy number of samples (copies/mL or copies/g). N is the average copy number of target genes in 20 μL ddPCR system, with three technical replicates performed for each sample (copies). V_1_ is the final constant volume of DNA extraction (μL). V_2_ is the volume of template DNA in ddPCR reaction system (μL), and B is the volume or mass of the homogenized sample used in DNA extraction (mL or g), d is the dilution coefficient of the sample during DNA extraction, for instance, if the sample is diluted at a ratio of 1:10, it would be designated as 10^−1^, M is the dilution factor of the DNA template before its addition to ddPCR.

### Quality control

An exogenous heterologous DNA fragment was used as extraction internal standard, which was spiked into each sample before lysis for co-extraction. The recovery rate of internal standard quantified by duplex ddPCR was applied to calibrate DNA loss during sample preprocessing. Extraction blank controls were prepared to exclude contamination. Duplex ddPCR simultaneously amplified target erm genes and extraction internal control to judge PCR inhibition. No-template control and positive plasmid control were included in each ddPCR batch to monitor contamination and verify amplification performance.

## Results

### Validation of primers and probes

**Figure 2:**
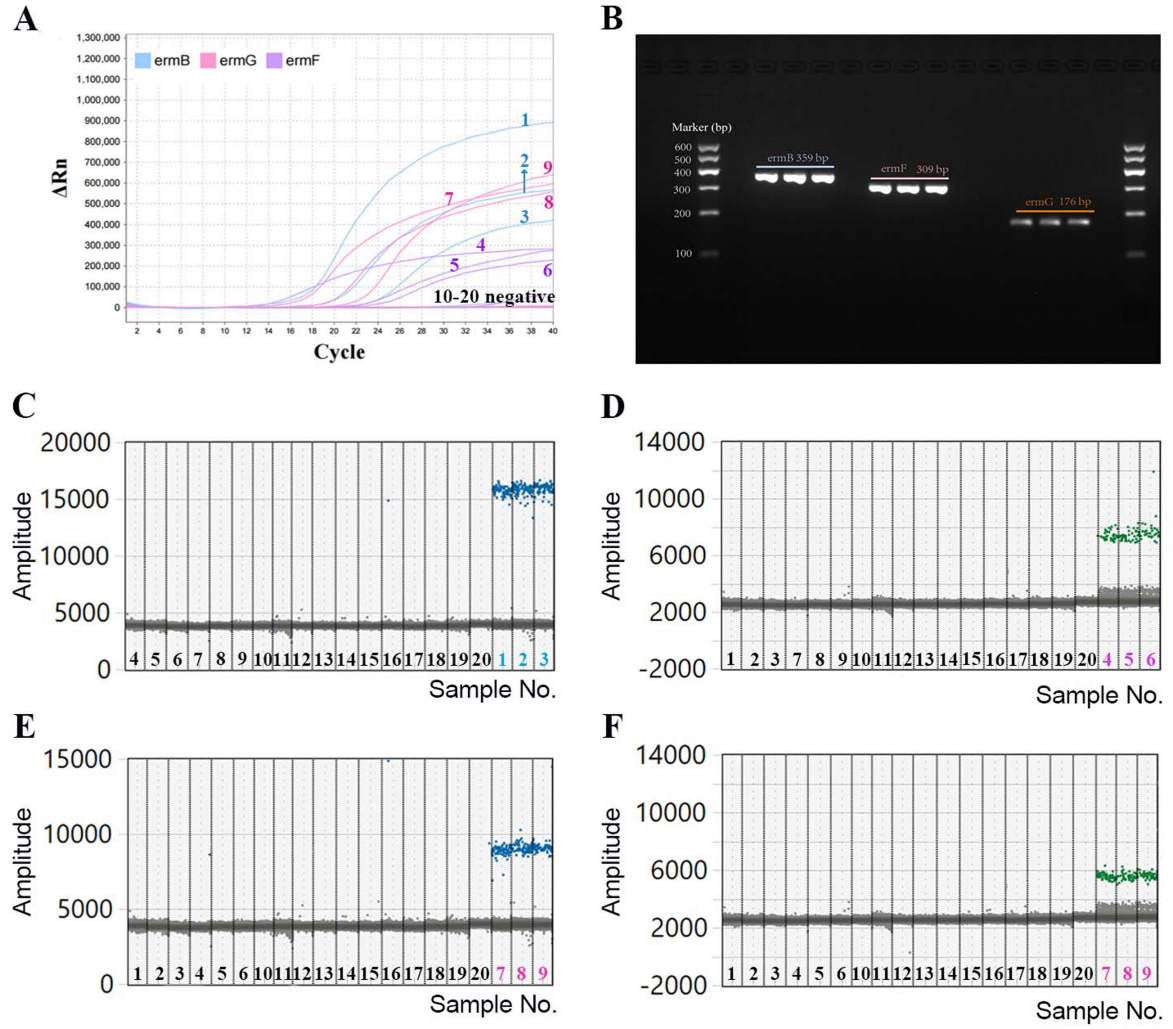
Comparison of the outcomes of PCR, qPCR and ddPCR in the detection of isolated strains. Among the 20 samples, samples numbered 1–3 are positive for *ermB,* samples 4–6 are positive for *ermF*, samples 7–9 are positive for *ermG,* 10–20 are negative samples. **(A)** The amplification curve of qPCR. **(B)** The agarose gel electrophoresis graph of PCR. **(C)** Detection results of *ermB* by single-target ddPCR. **(D)** Detection results of *ermF* by single-target ddPCR. **(E)** Detection results of *ermG* (FAM-labeled) by single-target ddPCR. **(F)** Detection results of *ermG* (HEX-labeled) by single-target ddPCR.

As depicted in Figure 2, 20 samples were detected using PCR, qPCR, and ddPCR methods. The results the three methods showed remarkable consistency. More precisely, three *ermB* - positive, three *ermF*—positive, three *ermG*—positive and 11 negative samples were identified, strongly validating the selectivity and specificity of the applied.

Subsequently, the PCR and qPCR products were sent to Beijing Tsingke Biotech Company (Beijing, China) for Sanger sequencing (ddPCR is a terminal detection technique, and its products cannot be recovered for sequencing). Finally, the sequencing results were compared with the reference sequence of *erm* genes by Snapgene software (Beijing, China). The sequence of PCR and qPCR products showed a high degree of matching with the reference sequence (Supplementary Figure 1), demonstrating that all primers and probes in the experiment successfully amplified the target gene fragment.

### Development of the triple ddPCR method

In the ddPCR method, droplets containing the target DNA sequences emit measurable fluorescence signals and are classified as positive droplets. Conversely, droplets without target sequences do not produce fluorescence and are designated as negative droplets. The fundamental requirement for achieving accurate and absolute quantification in ddPCR lies in the clear and unambiguous discrimination between positive droplet clusters, each representing specific target genes and the negative droplet cluster. This distinction is critical not only to differentiate the target presence from absence but also to resolve multiple targets in multiplex assays. Therefore, various experimental parameters that influence the fluorescence intensity of positive droplets must be rigorously optimized.

**Figure 3:**
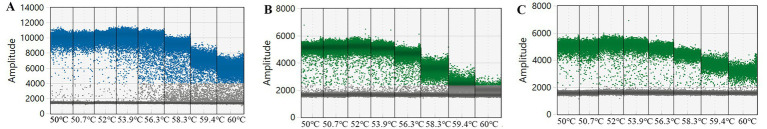
Optimization of annealing temperature for single ddPCR. **(A)** *ermB* gene; **(B)** *ermF* gene; **(C)** *ermG* gene.

The annealing temperature is a critical factor influencing the sensitivity, specificity, and overall success of droplet digital PCR (ddPCR) assays. In this study, we systematically optimized the annealing temperature to ensure efficient amplification and clear separation of droplet clusters for three target MLS_B_ resistance genes (*ermB*, *ermF*, and *ermG*). A temperature gradient ranging from 50 to 60 °C was programmed on a thermal cycler, covering eight discrete annealing temperatures. For each temperature condition, ddPCR was performed on reaction mixtures containing all three target gene probes and primers. The resulting one-dimensional (1D) fluorescence intensity plots were analyzed to evaluate the separation between positive and negative droplet populations. Higher annealing temperatures were found to suppress the fluorescence intensity of positive droplets, causing to clustering overlap and poor discrimination. This effect manifested as a compressed positive droplet cluster with fluorescence intensity’s approaching the negative cluster, thereby reducing assay sensitivity. Conversely, lower annealing temperatures promoted nonspecific amplification, generating “rainfall-like” droplets between positive and negative clusters on 1D plots, indicating partial or weak amplification signals. As illustrated in Figure 3, the fluorescence intensity of positive droplets for all three target genes decreased progressively with increasing annealing temperature, until the positive and negative clusters merged at the hignest tested temperature. Comprehensive analysis of the temperature gradient data indicated that the optimal annealing temperature range for the triplex ddPCR was 50–53.9 °C. To maximize assay stability and reproducibility across runs and instruments, 52 °C was selected as the final annealing temperature for all subsequent experiments.

**Figure 4:**
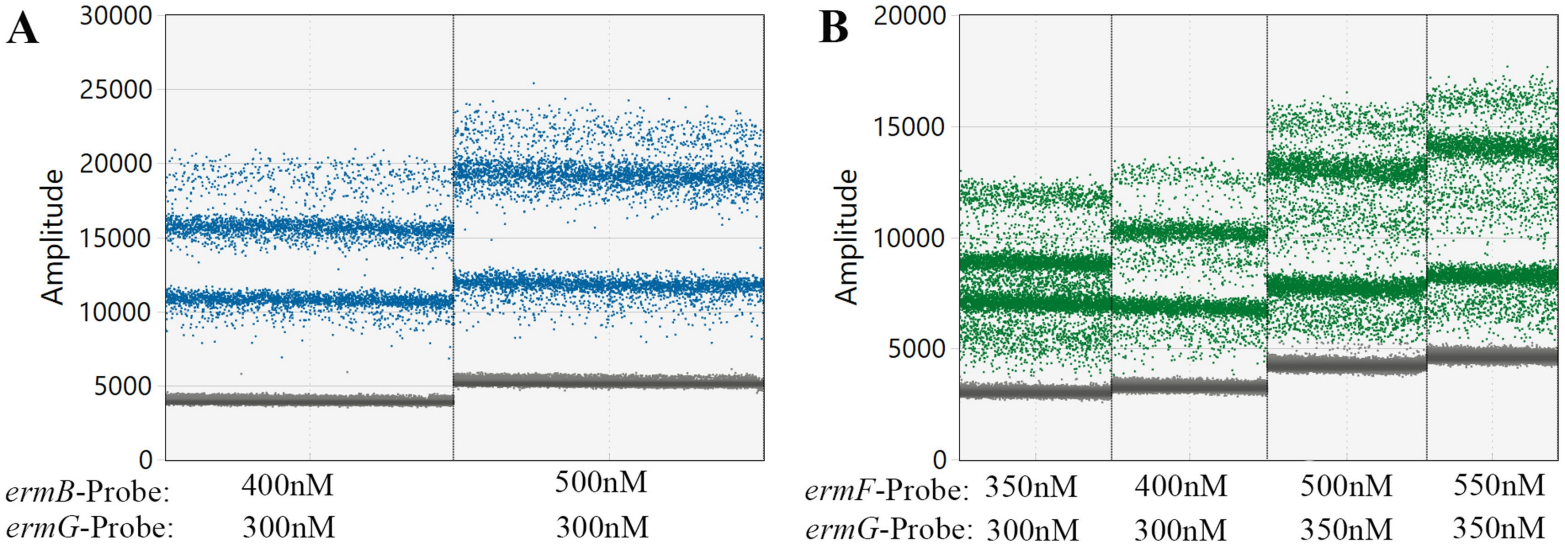
Optimization of probe concentration for triple ddPCR system. **(A)** Optimization of probe concentration for the FAM-labeled target. **(B)** Optimization of probe concentration for the HEX-labeled target.

To achieve simultaneous quantification of the three MLS_B_ resistance genes in a single ddPCR reaction, we employed the “probe ratio method” to design a multiplex assay compatible with the two-color detection system of the ddPCR platform (FAM and HEX channels). The initial primers/probe concentration ratio was 2:1 (Zhou et al., 2023; Cui et al., 2024). In this assay, *ermB* was labeled with FAM fluorescence, *ermF* was labeled with HEX fluorescence, and *ermG* detection utilized dual-labeled probes, one with FAM- and the other with HEX-labeled. Incorporating both FAM- and HEX-labeled probes for *ermG* within the reaction mixture results in droplets simultaneously exhibiting fluorescence signals in both channels. This unique dual fluorescence signature enabled the differentiation of *ermG* positive droplets from *ermB* or *ermF* positive droplets, which fluoresced in only one channel. Thus, this strategy allows unambiguous identification and quantification of all three target genes within a single multiplex assay. Probe concentration was identified as a key determinant influencing droplet fluorescence intensity and, consequently, the resolution of positive droplet clusters. Higher probe concentrations were associated with increased fluorescence intensity, enhancing cluster separation. To optimize this parameter, *ermB* and *ermF* probe concentrations were kept constant, while the concentration of *ermG* probes (both FAM- and HEX-labeled) was systematically titrated. As presented in Figure 4, demonstrate that an *ermB* probe concentration of 400 nM combined with an *ermG* probe concentration of 300 nM yielded the best separation of *ermB* and *ermG* droplet clusters in the FAM channel. Similarly, the optimal separation between *ermF* and *ermG* clusters in the HEX channel was achieved at 400 nM *ermF* and 300 nM *ermG* probe concentrations. Based on these observations, the final triplex ddPCR assay was configured with probe concentrations of 400 nM for *ermB* and *ermF*, and 300 nM for both FAM- and HEX-labeled *ermG* probes.

**Figure 5:**
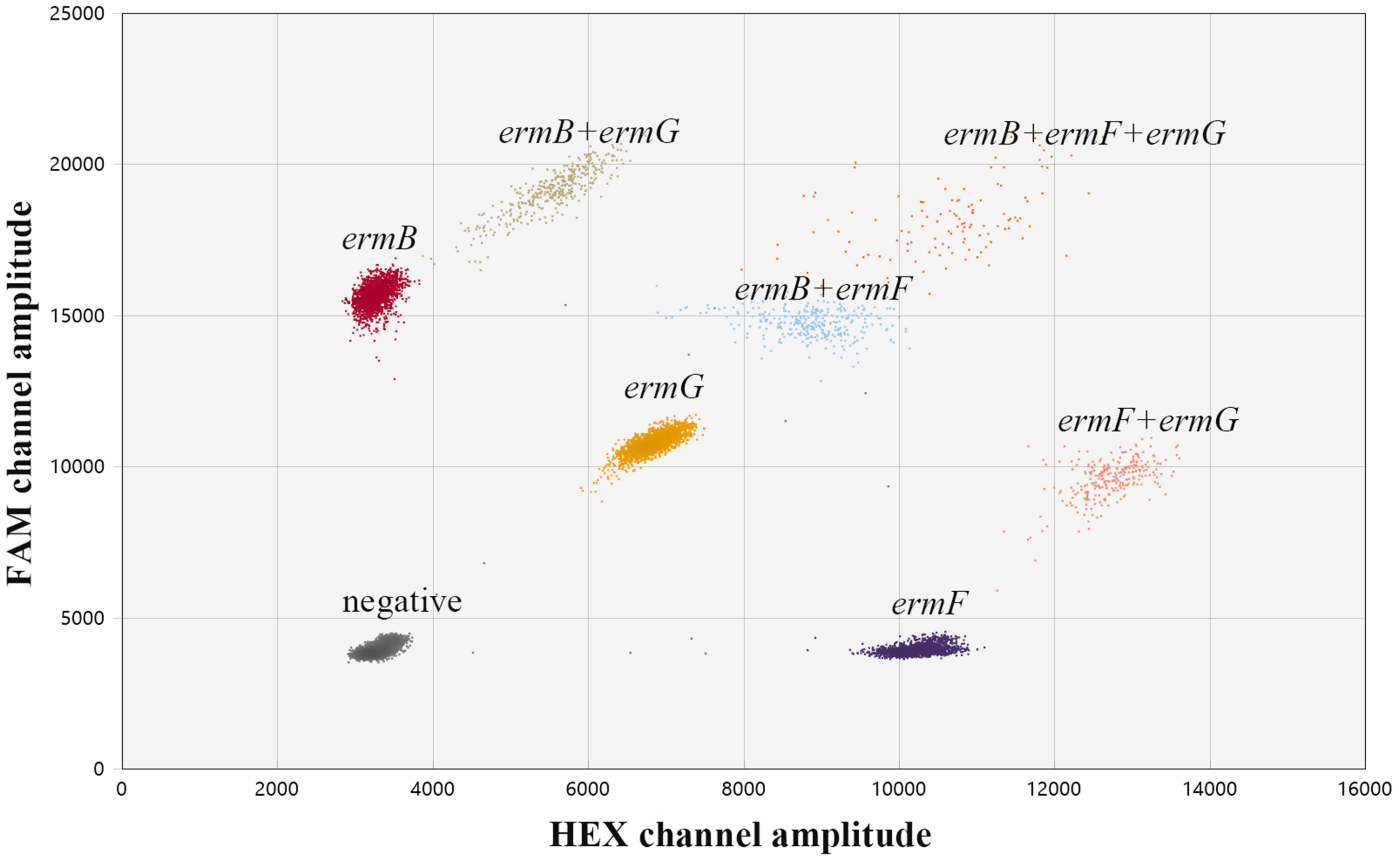
2D plot of the triple ddPCR.

The performance of the optimized triplex ddPCR assay was evaluated by assessing droplet cluster separation on two-dimensional (2D) fluorescence intensity plots, where fluorescence intensity in the HEX channel was plotted on the x-axis and that in the FAM channel on the y-axis. As depicted in Figure 5, the assay generated eight distinct droplet clusters, corresponding to all possible combinations of target gene presence or absence in individual droplets. Each cluster was assigned a unique color code for clarity, with the gray cluster representing negative droplets (no target gene amplification). The clear spatial segregation of these clusters confirms the assay’s capability to differentiate droplets positive for each single gene, as well as those positive for multiple targets due to probe co-localization.

The optimized triplex ddPCR assay demonstrated high precision and reproducibility, with well-resolved clusters enabling confident assignment of positive droplets to their corresponding targets. This multiplex approach significantly enhances throughput by reducing the number of reactions required for simultaneous detection of multiple MLSB resistance genes, while maintaining the sensitivity and specificity characteristic of ddPCR technology.

### Limits of detection, quantification range and repeatability of the triple ddPCR method

A mixed plasmid solution with a known concentration (5 × 10^1^ copies/μL) was serially diluted in fivefold steps to generate five concentration gradients. These diluted solutions were subsequently used as DNA templates in the triplex ddPCR assay, with each concentration tested in 12 replicates. The number of positive droplets at each concentration was recorded, and the limit of detection (LOD) along with the corresponding 95% confidence interval was calculated using a Probit regression model (Table 2).

**Table 2:** The LOD and 95% CI of ERM genes.

Genes	LOD (copies/reaction)	95% CI
*ermB*	4.82	(2.33, 5.81)
*ermF*	3.37	(2.18, 4.94)
*ermG*	8.31	(5.52, 10.12)

**Figure 6:**
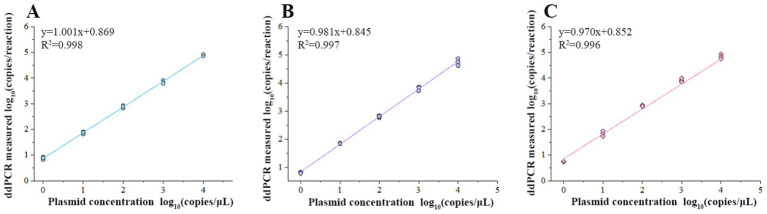
Quantification linearity of triple ddPCR: The X-axis represents the concentration of serially diluted linearized plasmid solutions and the Y-axis represents the detection results of triple PCR. The linear regression equation and the correlation coefficient (*R*^2^) are shown. **(A)** *ermB* gene; **(B)** *ermF* gene; **(C)** *ermG* gene.

To further determine the dynamic and reliable quantification range of the triplex ddPCR assay, plasmid mixtures covering five concentration gradients (from 10^0^ to 10^4^ copies/μL) were quantified using the same method. The measured values were plotted against the expected theoretical concentrations, followed by linear regression analysis. As illustrated in Figure 6, the x-axis represents the log_10_ of the plasmid input concentration, and the y-axis denotes the log_10_ of the measured copies per reaction obtained via the triplex ddPCR method (Table 3). All *erm* genes demonstrated strong linearity, with correlation coefficients (R^2^) exceeding 0.990.

**Table 3:** Plasmid solution for triple ddPCR detection of *erm* genes.

Genes	Concentration of plasmid DNA (copies/μL)	Triple ddPCR method		
		Copies/reaction ( $\bar{x}$ ± s)	CV(%)	95% CI
*ermB*	10^4^	(8.34 ± 0.72) × 10^4^	8.63	(6.55 × 10^4^, 1.01 × 10^5^)
	10^3^	(7.86 ± 0.53) × 10^3^	6.74	(6.54 × 10^3^, 9.18 × 10^3^)
	10^2^	(8.54 ± 0.37) × 10^2^	4.33	(7.62 × 10^2^, 9.46 × 10^2^)
	10^1^	(7.96 ± 0.42) × 10^1^	5.27	(6.92 × 10^1^, 9.00 × 10^1^)
	10^0^	(8.75 ± 1.61) × 10^0^	18.40	(4.75 × 10^0^, 1.27 × 10^1^)
*ermF*	10^4^	(7.44 ± 0.31) × 10^4^	4.17	(6.67 × 10^4^, 8.21 × 10^4^)
	10^3^	(7.18 ± 0.17) × 10^3^	2.32	(6.76 × 10^3^, 7.60 × 10^3^)
	10^2^	(6.96 ± 0.29) × 10^2^	4.33	(6.24 × 10^2^, 7.68 × 10^2^)
	10^1^	(7.48 ± 0.55) × 10^1^	7.35	(6.11 × 10^1^, 8.85 × 10^1^)
	10^0^	(6.84 ± 1.43) × 10^0^	20.80	(3.29 × 10^0^, 1.04 × 10^1^)
*ermG*	10^4^	(8.82 ± 0.47) × 10^4^	5.33	(7.65 × 10^4^, 9.99 × 10^4^)
	10^3^	(8.04 ± 0.64) × 10^3^	7.96	(6.45 × 10^3^, 9.63 × 10^3^)
	10^2^	(7.78 ± 0.37) × 10^2^	4.76	(6.86 × 10^2^, 8.70 × 10^2^)
	10^1^	(6.80 ± 0.51) × 10^1^	7.50	(5.53 × 10^1^, 8.07 × 10^1^)
	10^0^	(5.61 ± 1.28) × 10^0^	22.82	(2.43 × 10^0^, 8.79 × 10^0^)

### Detection of *erm* genes in diverse samples

**Figure 7:**
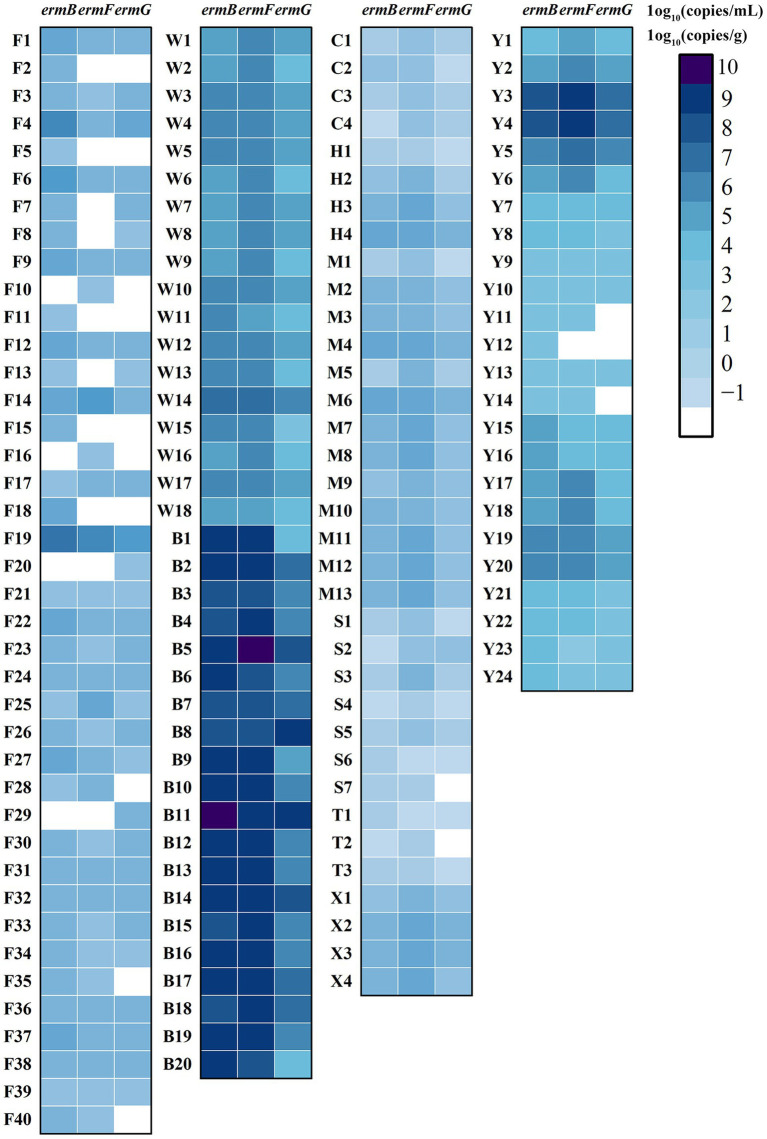
Logarithmic of *erm* gene concentration in different samples: F1 - F40 are animal-derived food samples; W1–W18 are sewage samples; B1–B20 are human feces samples; C1–C4 are surface water samples from Cha River; H1–H4 are surface water samples from Round-city water system; M1–M13 are surface water samples from Minxin River; S1–S7 are surface water samples from Shijin River; T1–T3 are surface water samples from Hutuo River; X1–X4 are surface water samples from South Flood Discharge Channel. Y1-Y24 are samples from livestock farms. For each sample, the higher the concentration, the darker the color, and white indicates no detection.

The triplex ddPCR method was applied to detect *erm* genes in 137 samples. Validation with 137 representative samples-including human feces, wastewater, surface water, food products, and livestock-related materials revealed high detection frequencies. Their average positive rate of *ermB, ermF, ermG* were 97.08, 91.97, and 88.32%, respectively (Table 4). To visualize the *erm* gene content in each sample, a logarithmic transformation was performed, with color intensity depicting the concentration level (Figure 7).

**Table 4:** The positive rates of triple ddPCR for *erm* genes in diverse samples.

Samples	*ermB*		*ermF*		*ermG*	
	Positive rates	95% CI	Positive rates	95% CI	Positive rates	95% CI
Animal-derived foods	90% (36/40)	76.95% ~ 96.04%	75% (30/40)	59.80% ~ 85.81%	72.50% (29/40)	57.16% ~ 83.89%
Sewage	100% (18/18)	82.41% ~ 100.00%	100% (18/18)	82.41% ~ 100.00%	100% (18/18)	82.41% ~ 100.00%
Human feces	100% (20/20)	83.89% ~ 100.00%	100% (20/20)	83.89% ~ 100.00%	100% (20/20)	83.89% ~ 100.00%
Surface waters	100% (35/35)	90.11% ~ 100.00%	100% (35/35)	90.11% ~ 100.00%	94.29% (33/35)	81.40% ~ 98.42%
farm-derived samples	100% (24/24)	86.20% ~ 100.00%	95.83% (23/24)	79.75% ~ 99.26%	87.50% (21/24)	69.00% ~ 95.65%
Average positive rate	97.08%	94.26% ~ 99.90%	91.97%	87.42% ~ 96.52%	88.32%	82.94% ~ 93.70%

**Figure 8:**
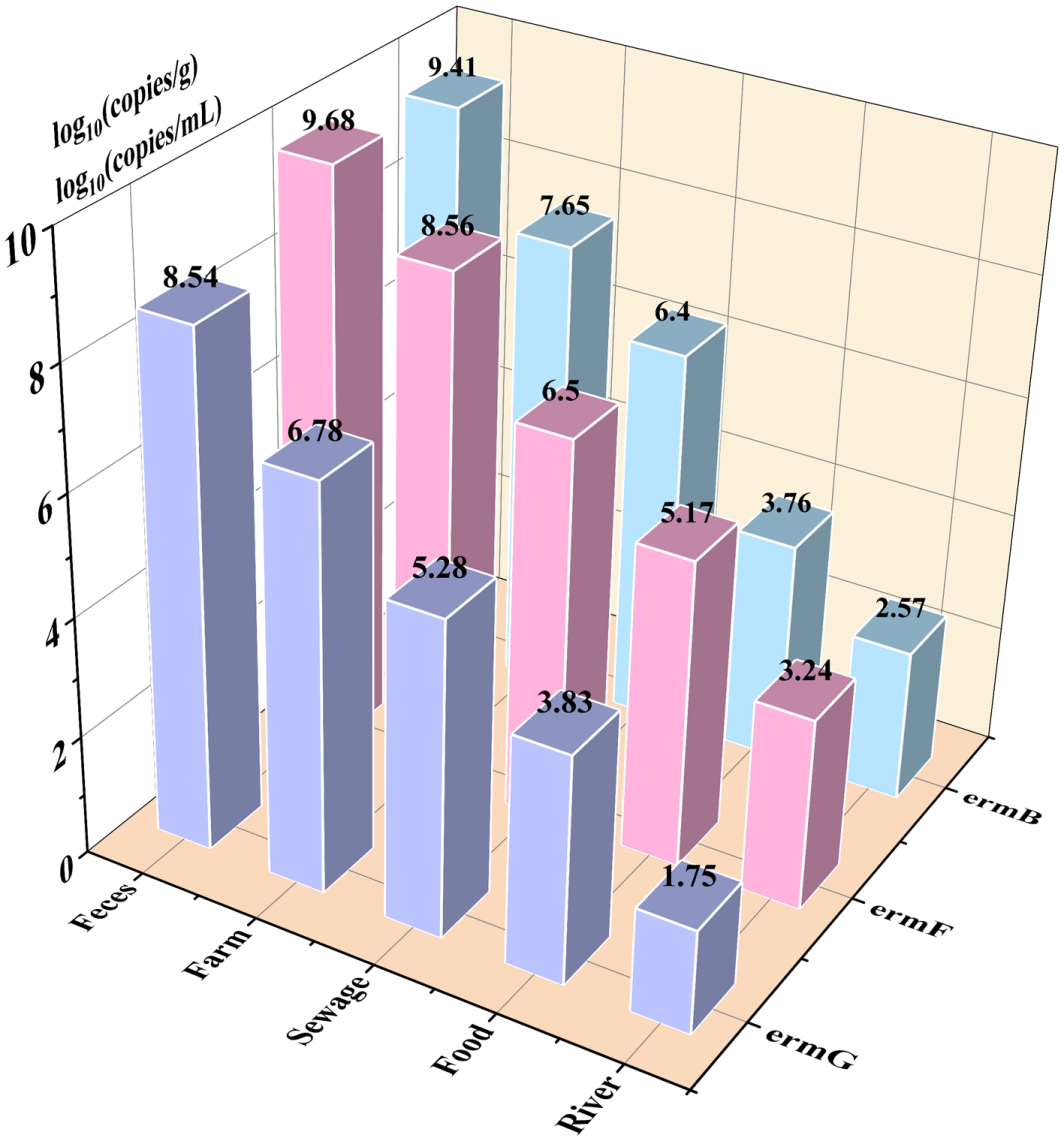
The logarithm of an average concentration of *erm* genes across different samples: the x-axis represents the sample types, the y-axis represents the *erm* gene names, and the z-axis represents the logarithmic values of average concentrations, log_10_ (copies/g) or log_10_ (copies/mL).

There were significant differences in the detection profiles of *erm* genes across different sample types. Facal samples exhibited the highest detection rates and concentrations of *erm* genes, followed by sewage samples. In food products, the detection rates of *erm* genes varied widely, suggesting potential differences in gene abundance among animal-derived foods and even tissue types within the same category. Notably, farm-derived samples-particularly Y3-Y6 and Y19-Y20 (bovine and chicken fecal samples)-showed significantly elevated *erm* gene levels compared to others. In surface water, while *erm* detection rates were relatively high, concentrations were comparatively low, indicating diverse MLS_B_ resistant bacteria populations at lower abundances than sewage. To further explore *erm* gene concentration differences, average levels of the three *erm* genes were calculated across five sample categories (Figure 8). Human feces had the highest average *erm* concentrations, followed by sewage, anima-derived foods, and surface water. *ermB* and *ermF* in human feces reached extremely high levels (1.29 × 10^10^ copies/g and 4.37 × 10^7^ copies/g, respectively). Compared with other types of samples, they were 1–4 orders of magnitude higher. Sewage samples also harbored abundant *erm* genes (3.89 × 10^5^ to 4.47 × 10^7^ copies/mL), while animal-derived foods showed moderate concentrations (1.7 × 104 to 2.75 × 10^6^ copies/g), significantly lower than sewage and feces. Surface water had the lowest *erm* concentrations (3.55 × 10^2^ to 1.70 × 10^5^ copies/mL). Across all samples, *ermB* and *ermF* average concentrations (9.78 × 10^6^ copies/g and 5.75 × 10^6^ copies/g, respectively) far exceeded *ermG* (2.40 × 10^4^ copies/g and 5.49 × 10^4^ copies/g respectively). In this study, the concentration distribution of *erm* genes exhibited a pattern of highest abundance in fecal samples, followed by untreated sewage, and lowest in surface water and food samples, which is consistent with the concentration gradients of ARGs reported in studies (Wang X. et al., 2022; Wu et al., 2025; Williams et al., 2023; Abramova et al., 2023).

In summary, *erm* gene concentrations correlated with microbial abundance, peaking in human feces and sewage. The observed disparities in detection rates and concentrations across sample types. It is necessary to strengthen the monitoring of *erm* genes and explore the potential reasons of these distinctions.

## Discussion

No single method is superior to ddPCR in all scenarios. Whole-genome sequencing covers comprehensive resistance genes and strain tracing but is costly and unsuitable for accurate absolute quantification (Napit et al., 2025). Isothermal CRISPR assays are cheap and rapid for on-site screening yet lack reliable quantification (Zhang et al., 2024). High-throughput qPCR detects multiple targets at low cost, but its relative quantification is easily disturbed by sample inhibitors (Ben Maamar et al., 2020). For absolute quantification of a few resistance genes in complex samples, ddPCR is still the best option (Larocque et al., 2022).

The triplex ddPCR method developed in this study provides robust technical support for the standardized surveillance of three MLSB resistance genes (*erm*B, *erm*F, and *erm*G), that exhibit distinct ecological distribution patterns. By leveraging the inherent advantages of droplet digital PCR, including precise absolute quantification without reliance on standard curves and robust tolerance to PCR inhibitors, this method addresses critical limitations of conventional qPCR approaches. As summarized in Table 5, the limit of detection achieved by our platform demonstrates superior sensitivity compared to previously reported methods, enabling reliable detection of low-abundance targets in diverse complex matrices.

**Table 5:** The comparison of this study with other methods for quantification of ERM genes.

Author (year)	Detection method	*erm* genes	Sensitivity	Sample type
Chen et al. (2007)	qPCR	*ermA, ermB ermC, ermF, ermT, ermX*	88.6 ~ 4.18 × 10^3^ copies/reaction	Bovine manure, swine manure, compost of swine manure, swine waste lagoons
Neher et al. (2023)	qPCR	*ermF*	50 copies/reaction	Swine manure
Mao et al. (2024)	CRISPR/Cas12a and LAMP	*ermB*	2.75 × 10^3^ copies/μL	Wastewater
Gobbo et al. (2024)	ddPCR	*ermB*	13 copies/reaction	Wastewater
This study	ddPCR	*ermB, ermF, ermG*	3.37 ~ 8.31 copies/reaction	Feces, Sewage, Surface waters, Animal-derived foods, farm-derived samples

One of the key advantages of ddPCR technology lies in its ability to partition samples into thousands of nanoliter-scale droplets, enabling absolute quantification of target DNA molecules without reliance on external standards or calibration curves (Cheng et al., 2024). This partitioning also reduces the impact of PCR inhibitors frequently encountered in complex environmental and biological matrices (Alcaide and Morin, 2018). In our triplex assay, the integration of three target genes into a single reaction not only improved analytical throughput but also maintained high sensitivity and specificity, as reflected by detection limits reaching single-digit copy numbers (Cui et al., 2020). Compared to traditional quantitative PCR (qPCR), ddPCR is less susceptible to amplification efficiency variations, providing more precise and reproducible quantification, especially at low target concentrations (Ben Maamar et al., 2020). This is particularly important when tracking resistance genes in environmental reservoirs where gene copy numbers may fluctuate widely or exist at very low abundance (de la Cruz Barron et al., 2023). The ability of our method to detect *ermB*, *ermF*, and *ermG* with detection frequencies above 80% across 137 diverse samples, including human feces, wastewater, surface water, food, and livestock-related samples.

The high detection rates of *ermB*, *ermF*, and *ermG* observed in this study highlight the widespread presence of MLS_B_ resistance genes across multiple environmental and host-associated compartments. Notably, human fecal and wastewater samples exhibited the highest positivity rates and gene concentrations, underscoring these compartments as critical reservoirs and potential sources of MLS_B_ resistance dissemination. In contrast, river water is a diluted environmental matrix. Fecal pollutants are continuously diluted by large volumes of water, and ultraviolet irradiation, microbial degradation, adsorption by sediment, and nutrient limitation in the aquatic environment jointly reduce the absolute abundance of *erm*-carrying bacteria and free resistance DNA, resulting in significantly lower gene copy numbers in river samples. This distinct distribution pattern highlights fecal waste as a critical reservoir of erm resistance genes, with surface water serving as a diluted transmission medium for such antimicrobial resistance determinants. This observation aligns with previous studies identifying wastewater treatment plants and human gut microbiota as hotspots for ARGs proliferation and horizontal gene transfer (Curiao et al., 2011; Kirschner et al., 2024).

Quantitative analysis revealed that *ermB* and *ermF* genes were more abundant than *ermG* across all sample types, with *ermF* concentrations reaching as high as 1.29 × 10^10^ copies per gram in fecal samples. The predominance of *ermB* and *ermF* may be attributable to their association with mobile genetic elements such as plasmids and transposons, which facilitate their rapid spread among diverse bacterial population (Rong et al., 2021). These findings reinforce the critical need for monitoring multiple MLS_B_ genes simultaneously to fully capture the resistance landscape and avoid underestimation of varied matrices demonstrates its potential as a valuable tool for environmental and public health surveillance of MLS_B_ resistance genes (Sarrou et al., 2019). Precise quantification of ARGs can inform risk assessments, guide interventions, and evaluate the effectiveness of mitigation strategies aimed at reducing AMR dissemination. For instance, tracking the abundance and distribution of MLS_B_ genes in wastewater treatment systems can help optimize treatment processes to minimize ARG release into receiving waters (Gobbo et al., 2024).

Furthermore, the capability to simultaneously quantify multiple target genes within a single assay reduces sample processing time, reagent consumption, and overall costs, enhancing the feasibility of large-scale monitoring programs (Guo et al., 2017) This approach can be readily adapted to include additional resistance markers or virulence genes, broadening its utility in comprehensive AMR surveillance frameworks While the triplex ddPCR assay offers significant improvements in sensitivity and multiplexing capacity, several methodological considerations should be noted. First, sample preprocessing and DNA extraction protocols must be carefully optimized to minimize inhibitors and maximize DNA recovery, as variations can influence quantification accuracy. In this study, samples were uniformly stored at −80 °C prior to extraction to preserve nucleic acid integrity, and extraction procedures were standardized to ensure consistency. Second, despite the high specificity of the designed primers and probes, potential cross-reactivity with closely related gene variants or environmental sequences cannot be entirely ruled out. Therefore, sequencing validation or complementary methods such as metagenomic analysis may be employed to confirm ddPCR results when novel or unexpected detection patterns arise (Davis et al., 2025). Finally, while absolute quantification provides valuable data on gene copy numbers, it does not directly indicate gene expression levels or phenotypic resistance. Integrating ddPCR data with transcriptomic or culture-based assays would offer a more comprehensive understanding of resistance gene functionality and clinical relevance (Quezada-Aguiluz et al., 2022; Wang Y. T. et al. 2022).

Building on the robust foundation established in this study, several future directions merit consideration. Expanding the multiplex panel to include additional MLS_B_ resistance genes or other ARG families would further enhance surveillance capabilities and provide a holistic view of the resistome in environmental and clinical settings. Integration of high-throughput automation and microfluidic technologies could facilitate rapid, large-scale screening with minimal hands-on time (Rong et al., 2021). Moreover, coupling ddPCR quantification with spatial and temporal monitoring will enable the assessment of resistance gene dynamics in response to anthropogenic pressures such as antibiotic usage, agricultural practices, or wastewater treatment interventions. Such longitudinal data are critical for modeling resistance gene dissemination pathways and predicting emerging threat (Sarrou et al., 2019). Lastly, the applicability of this method extends beyond environmental microbiology into clinical diagnostics and epidemiology, where rapid, sensitive, and multiplexed detection of resistance genes can inform patient management and infection control measures (Federigi et al., 2024).

DNA loss is inevitable during sample preprocessing and DNA extraction procedures. High-efficiency nucleic acid extraction kits are therefore adopted to mitigate these systematic errors. Owing to the intrinsic limitations of PCR detection, positive amplification of target gene fragments does not equate to viable bacteria carrying resistant phenotypes, which necessitates bacterial isolation and culture assays for phenotypic verification. This work aims to develop a triplex digital PCR assay and validate its performance across various sample matrices. For comprehensive environmental epidemiological surveillance, larger sample cohorts, more sampling batches, and supplementary genomic sequencing analyses are required to fully elucidate the prevalence characteristics of antimicrobial resistance genes.

In conclusion, the development of a highly sensitive triplex ddPCR assay for the simultaneous quantification of *ermB*, *ermF*, and *ermG* genes represents a significant advancement in molecular tools for *erm* genes monitoring. The new method whith superior sensitivity, precision, and multiplexing ability enable comprehensive profiling of MLS_B_ resistance genes across diverse sample types, revealing their widespread prevalence and high abundance in key environmental reservoirs. These capabilities pave the way for improved surveillance, risk assessment, and ultimately, more effective strategies to combat the global challenge of antimicrobial resistance.

## Conclusion

A highly precise, reliable, and sensitive triple ddPCR method for detecting *ermB*, *ermF,* and *ermG* genes simultaneously was developed in this study. This novel approach integrates the advantages of advanced ddPCR, enabling absolute quantification of MLS_B_ genes in a wide range of complex sample matrices, with a limit of detection as low as single-digit copy numbers. Subsequently, application of the method to 137 diverse samples, comprising human feces, wastewater, surface water, food, and livestock-related materials, which yielded positive detection rates above 80% for all three *erm* genes (*ermB*, *ermF*, and *ermG*). Obviously, the results demonstrated the applicability and high sensitivity across complex matrices of the new method. Collectively, these findings highlight the feasibility and value of triplex ddPCR for routine monitoring of MLS_B_ resistance genes. Wider deployment of this method will advance the mechanistic understanding of resistance dissemination and inform evidence-based measures to curb the environmental spread of MLS_B_ resistance issues. This study complied with the dMIQE guidelines (Minimum Information for Publication of Quantitative Digital PCR Experiments for 2020).

## Data Availability

The original contributions presented in the study are included in the article/Supplementary material, further inquiries can be directed to the corresponding authors.
