# Supplementary material for: Development and application of a triplex ddPCR method for simultaneous detection of MLSB resistance genes in feces and environmental samples
Source: Front Microbiol. 2026 Aug 5;17:1887402. doi: 10.3389/fmicb.2026.1887402 (PMC13485974; doi:10.3389/fmicb.2026.1887402)
Supplement: Supplementary file 1 [file Table_1.pdf]

Supplementary Material

Development and application of a triplex ddPCR method for Simultaneous detection of MLS_B resistance genes in feces and environmental samples

1. The experimental procedure of PCR

The reaction system of PCR consists of 25 µL of 2×TaKaRa Taq HS Perfect Mix (Takara Bio Inc, Japan), 5 µL of primers (10µM) and 5 µL of template DNA, and 10 µL nuclease-free water is supplemented to make a total volume of 50 µL. Amplification conditions, heating for 3 min at 94 ℃, followed by 30 cycles at 94 ℃ for 5 s, hold for 5 s under the appropriate annealing temperature, and 68 ℃ for 20 s. The primers and experimental conditions of the first-generation PCR are presented as follows. (Supplementary Table 1).

Supplementary Table 1. The primers of PCR

Gene	Primer	Sequence 5'- 3'	Product size(bp)	annealing temperatue (℃)	References
ermB	*ermB*-F	CCGTTTACGAAATTGGAACAGG TAAAGGGC	395	55	Trieu-Cuot et al. 1990
	ermB-R	GAATCGAGAC TTGAGTGTGC			
ermF	*ermF*-F	CGACACAGCTTTGGTTGAAC	309	56	Tian et al. 2019
	ermF-R	GGACCTACCTCATAGACAAG			
ermG	*ermG*-F	ATAGGTGCAGGGAAAGGTCA	176	59	Rong et al. 2021
	ermG-R	TGGATTGTGGCTAGGAAATGT			

2. Development of triplex qPCR approache.

According to the melting temperature of the primers and probes (Supplementary Table 1), a series of temperature gradients were set up to determine the optimal annealing temperature of each target. Then, the primers and probes of the three target genes were combined pairwise for qPCR amplification to confirm no mutual interference. Later, different combinations of primers and probes were designed, the optimal multiplex qPCR system was selected based on a lower Ct value and a higher fluorescence intensity in the amplification curve. The methodology is presented as follows:The multiplex qPCR mixture contained 10µL 2× Premix Ex Taq (Takara Bio Inc, Japan), 0.8 µL each primer (10 µM), 0.3 µL *ermB* probe (10 µM,FAM-labled), 0.4 µL *ermF* probe (10 µM, HEX-labled), 0.3 µL *ermG* probe (10 µM, ROX-labled), 2 µL DNA sample and 2.2 µL nuclease-free water to a final volume of 20 µL. All reactions were amplified on a Applied BiosystemsTM 7500

Real-time System (Thermo Scientific, USA) at 95℃ for 30 s, followed by 40 cycles of 95℃ for 5 s and 52℃ for 30 s

The standard curves of *erm* genes were generated using the gradient-diluted plasmid mentioned above, the best linear fitting effect is in the range of 107 to 101 copies/μL. Ideal qPCR methods demand that the correlation coefficient(R2) > 0.990, the slope of the standard curve ranges from -3.1 to -3.6, and the amplification efficiency range (Eff%) between 90%~110%. The multiplex qPCR system in this study fully complies with the requirements (supplementary Fig 1).

Supplementary Table 2. The primers and probes for triplex qPCR.

Genes	Gene bank number and position	Primer/ Probe	Sequnence (5'- 3')	Product size (bp)	Refer nce
ermB	AF242872.1; 2132-2878	*ermB*-F	ACGGCCAACACAATAGGT	83 bp	This study
		ermB-R	GGATTCTACAAGCGTACCTTGGA		
		ermB-P	FAM-CACTAGGGTTGCTCTTGCACACTCAAGTC-BHQ1		
ermF	M17124.1; 1182-1982	*ermF*-F	GAATTTTGCAGTTCCGAA	80 bp	This study
		ermF-R	GGATTTTGAAAATATCGGAAG		
		ermF-P	HEX-ATGCCATAAGGAATATTTGACACC-BHQ1		
ermG	L42817.1; 202-936	*ermG*-F	ACATCTTTGAAATAGGTGC	86 bp	This study
		ermG-R	ATCAATTTCTATCGCCGTA		
		ermG-P	ROX-AAGGTCATTTTACTGCTGAATTGG-BHQ2		

F, forward primer; R, reverse primer; P, probe; FAM, fluorescein amidite; HEX, hexachloro fluorescein; ROX, carboxy-X-rhodamine; BHQ1, black hole quencher 1; BHQ2, black hole quencher 2. FAM, HEX, and ROX are reporter groups that modify probes, while BHQ1 and BHQ2 are quencher groups that modify probes.

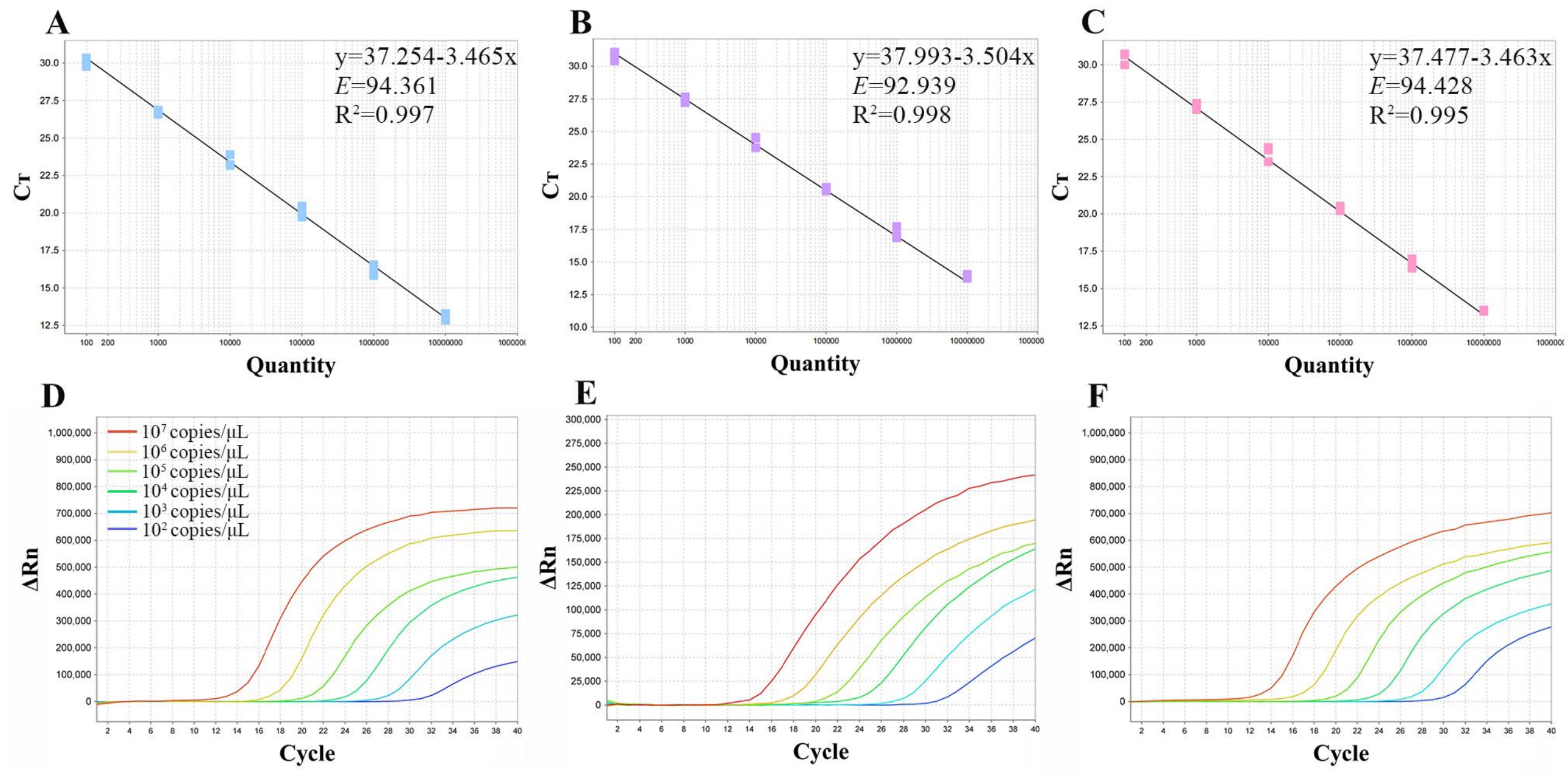

Supplementary Figure 1. Standard curve and amplification curve of triplex qPCR: (A) *ermB* gene; (B) *ermF* gene; (C) *ermG* gene; (D) *ermB* gene; (E) *ermF* gene; (F) *ermG* gene.

References

Rong, S. M. M., Rodloff, A. C., and Stingu, C. S. (2021). Diversity of antimicrobial resistance genes in Bacteroides and Parabacteroides strains isolated in Germany. *J Glob Antimicrob Resist* 24, 328-334. doi: 10.1016/j.jgar.2021.01.007.

Tian, J., Wu, N., Xie, S., Huang, Z., and Yuan, S. (2019). Analysis of ermF gene host bacterial diversity in soil based on high-throughput sequencing. *Tianjin Agricultural Sciences (China)*.25(11) 53-56.

Trieu-Cuot, P., Poyart-Salmeron, C., Carlier, C., and Courvalin, P. (1990). Nucleotide sequence of the erythromycin resistance gene of the conjugative transposon Tn1545. *Nucleic Acids Res* 18, 3660. doi: 10.1093/nar/18.12.3660.
